# YouTube videos on lymphedema as an information source for Spanish speaking breast cancer survivors

**DOI:** 10.1007/s00520-024-08746-2

**Published:** 2024-07-24

**Authors:** Maria del Mar Fernandez-Alvarez, Judit Cachero-Rodríguez, Mei Rosemary Fu, Paula Sánchez-Fernández, Amalia Ureña-Lorenzo, Ruben Martin-Payo

**Affiliations:** 1 Facultad de Medicina y Ciencias de La Salud, Universidad de Oviedo, Campus del Cristo S/N, 33006 - Oviedo, Asturias, Spain; 2 Equipo de Investigación Precam, Instituto de Investigación Sanitaria del Principado de Asturias, Oviedo, Asturias Spain; 3 The Dorothy and Dale Thompson Missouri Endowed Professor in Nursing and Associate Dean for Research, University of Missouri-Kansas City, School of Nursing and Health Studies, Kansas City, MO USA; 4 Servicio de Salud del Principado de Asturias, Área Sanitaria 3, Avilés, Spain

**Keywords:** Lymphedema, Breast cancer, YouTube, Patient education, Internet

## Abstract

**Background:**

Breast cancer-related lymphedema in the upper limb remains one of the most distressful complications of breast cancer treatment. YouTube is considered a potential digital resource for population health and decision making. However, access to inadequate information or misinformation could have undesirable impacts. This cross-sectional study aimed to evaluate the reliability, quality and content of YouTube videos on lymphedema as an information source for Spanish-speaking breast cancer survivors.

**Methods:**

A search of YouTube was conducted in January 2023 using the key words “breast cancer lymphedema” and “lymphedema arm breast cancer.” Reliability and quality of the videos were evaluated using the Discern tool, content, source of production, number of likes, comments, views, duration, Video Power Index, likes ratio, view ratio and age on the platform.

**Results:**

Amongst the 300 Spanish language videos identified on YouTube, 35 were selected for analysis based on the inclusion and exclusion criteria. Of the 35 selected videos, 82.9% (*n* = 29) were developed by healthcare or academic professionals and 17.1% (*n* = 9) by others. Reliability (*p* < 0.017) and quality (*p* < 0.03) were higher in the videos made by professionals. The Discern total score (*r* = 0.476; *p* = 0.004), reliability (*r* = 0.472; *p* = 0.004) and quality (*r* = 0.469; *p* = 0.004) were positively correlated with the duration of the videos.

**Conclusions:**

Our findings provide a strong rationale for educating breast cancer survivors seeking lymphedema information to select videos made by healthcare or academic professionals. Standardised evaluation prior to video publication is needed to ensure that the end-users receive accurate and quality information from YouTube.

**Supplementary Information:**

The online version contains supplementary material available at 10.1007/s00520-024-08746-2.

## Introduction

At least one in five of more than 7.8 million women treated for breast cancer worldwide are affected by lymphedema [1, 2]. Lymphedema in the upper arm remains one of the most distressful and long-term complications of breast cancer treatment [3] and negatively impacts the quality of life of breast cancer survivors [4, 5]. Lymphedema is characterised by the presence of swelling, pain, aching, soreness, tenderness, heaviness or impaired movement in the affected upper limb [6, 7]. Research evidence demonstrates that lymphedema information is essential for breast cancer survivors to reduce the risk of and manage lymphedema [8]. Several investigations have shown that providing information about lymphedema and self-care has positive effects on the improvement of lymphedema symptoms and the adoption of healthy behaviours [9–12]. Such findings are supported by the Behaviour Change Wheel Model [BCW] [13] that emphasises the positive effects of educational strategies on the psychological capacity of individuals and reflective motivation to promote behavioural changes. Despite the positive effects of providing lymphedema information and the long-term adverse impacts of lymphedema on breast cancer survivors, several studies have shown that a considerable percentage of survivors claim not to receive adequate information about lymphedema from health professionals [14–16]. This often leads breast cancer survivors to search for information from other sources, such as the Internet [17] or social networks [18, 19].

YouTube is the second most visited website in the world after Google [20, 21], and is considered to be a digital resource that may have a potential impact on population health behaviours and decision making. As such, YouTube may be a potential digital resource for breast cancer survivors seeking lymphedema information. Given that the information contained on YouTube is not subject to a review process by experts [22, 23], videos may contain inaccurate information or misinformation that affects their effectiveness, scientific quality and reliability [24, 25]. In addition, inaccurate or misleading content may produce harmful or ineffective effects on the promotion of healthy behaviours (e.g., behaviours to prevent or manage lymphedema) [26]. Most importantly, a video with misleading content can mislead viewers into wrong decisions or behaviours (e.g., dietary habits) [27]. For example, women with breast cancer should be educated about and encouraged to follow healthy dietary habits recommended by experts [28], since excess body weight increases the risk of developing lymphedema [29]. Singh et al. [30] and Ayoub et al. [31] identified the most common misleading videos including information about therapies that are scientifically inaccurate, or lack research evidence, and respectively concluded that 30.4% and 42% of the YouTube videos could be classified as misleading. The most common misleading videos include information that is scientifically unproven or inaccurate, for example, unscientific therapies [26, 27]. Several studies have provided evidence that inadequate or misleading content on YouTube can be harmful, after having evaluated the quality of YouTube content regarding various chronic illnesses such as fibromyalgia [32], rheumatoid arthritis [30], endometriosis [33] or breast cancer [31, 34, 35], as well as other chronic diseases [36–38].

Nevertheless, YouTube has been recognised as a tool for disseminating health information. According to Norgaard et al. [39], YouTube encompasses system features for digital services that do not require users to have specific or high-level skills to search and access information based on individual needs. These features make YouTube a popular digital service with the potential to impact public health in the digital era.

The accessibility to health-related content on YouTube and women’s desire for information suggest that survivors will seek information in web-based contexts, including YouTube. The lack of professional or expert evaluation of YouTube sources concerning lymphedema implies that women have a high chance of finding health information that is inaccurate, false or inadequate. With the sheer number of videos available, it is essential to establish professional criteria to systematically evaluate videos on health issues, such as lymphedema following breast cancer treatment, in order to determine which videos should be recommended to patients. Such systematic evaluation of videos on health issues such as lymphedema in Spanish is critical, as Spanish is the fourth most commonly spoken language in the world, and currently there are 22 Spanish-speaking countries worldwide [40]. In addition, patient education on lymphedema prevention and treatment is usually not considered part of clinical practice, and many patients still have not received any education on lymphedema risk reduction and treatment from health providers [14–16]. This is also true for Spanish-speaking breast cancer survivors, and these patients certainly seek lymphedema information on YouTube [17–19]. To date, no studies have been conducted to evaluate the adequacy of YouTube videos on lymphedema following breast cancer treatment in the Spanish language, and there is an urgent need to assess whether YouTube Spanish language videos on lymphedema are adequate for health professionals to recommend them to patients for the prevention and management of upper-limb lymphedema related to breast cancer. Thus, the purpose of this study was to assess the characteristics, content, quality and reliability of YouTube videos on lymphedema in the Spanish language for breast cancer survivors in preventing and treating lymphedema in the upper limbs.

## Methodology

### Design

The present study comprised a cross-sectional design with a two-step process to (i) identify relevant YouTube videos in the Spanish language focused on the prevention or treatment of breast cancer-related lymphedema in the upper limbs; and (ii) assess the characteristics, content, quality and reliability of the selected videos. No human subjects were involved in the study.

#### Step One: Identification and selection of relevant videos

A simulation method was used to imitate the way in which a patient would access YouTube to try to find videos providing information on breast cancer-related lymphedema prevention or treatment. In January 2023 a search of YouTube was performed to identify Spanish language videos on breast cancer-related lymphedema using the following keywords in Spanish: “linfedema cáncer de mama” (breast cancer lymphedema), “linfedema brazo cáncer de mama” (lymphedema arm breast cancer). The searches resulted in 300 videos that were listed based on the rank of relevance of the YouTube videos [41].

Inclusion criteria for the selection of videos were: i) content focusing on breast cancer-related lymphedema in the upper limbs; ii) free access; and iii) availability in Spanish. Exclusion criteria were: i) advertisement videos; ii) video malfunction; and iii) content focusing on other forms of lymphedema, including leg lymphedema, congenital lymphedema or filariasis. Two researchers conducted the initial assessment of the 300 videos to eliminate 100 duplicated videos and evaluate the eligibility of 200 videos based on the inclusion and exclusion criteria. The initial assessment yielded 35 videos for the systematic evaluation.

#### Step Two: Assessment of the characteristics, content, quality and reliability of selected videos

**Fig. 1:**
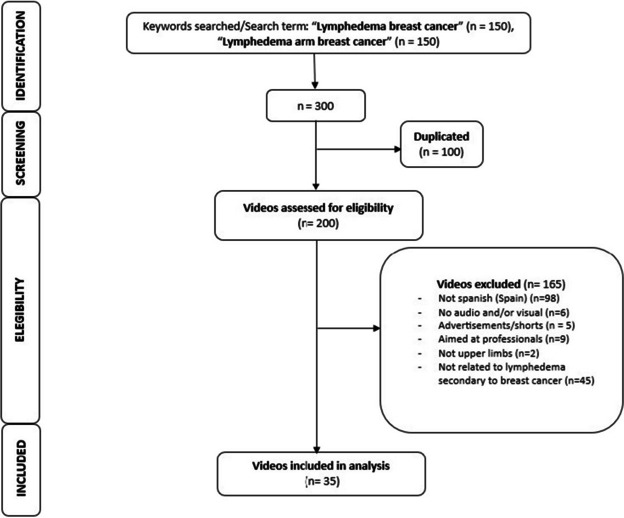
Flowchart of app search process

Two researchers independently evaluated the 35 selected videos systematically for characteristics, content, quality and reliability (Fig. 1). Any discrepancies between the evaluators were resolved by discussion with a third researcher to reach a consensus.

##### Characteristics

The description of the characteristics of the videos included the number of likes, number of comments, number of views, duration of the video (in minutes), year of upload to the platform, time elapsed from upload to analysis (days), and popularity using the Video Power Index (VPI). This index was calculated using the formula (likes ratio × view ratio) / 100 [42]. In previous research [33], dislikes were included for the evaluation; however, due to the recent change in YouTube policy, the videos do not publicly display the number of dislikes and, therefore, dislikes could not be used as an indicator for the characteristics in this study.

##### Content

The videos were classified according to content and source of production [20, 32–34, 37]. The videos were categorised into 5 groups based on their content: (1) informative videos, explaining lymphedema, its aetiology, symptoms and/or diagnosis; (2) preventive videos, focusing on the prevention of lymphedema; (3) treatment videos, focusing on different lymphedema treatment options; (4) informative and preventive (I + P) videos; and (5) preventive and treatment (P + T) videos. The videos in turn were classified based on the source of production as videos made by healthcare/academic professionals, or by others, such as patients (e.g., personal experiences) or commercial entities (brands of health products).

##### Video quality and reliability

The Discern tool was used to assess quality (i.e., content accuracy) and reliability (i.e., content consistency) of the videos [43]. The Discern tool was published in 1998 to facilitate health decision-making [43]. Discern has been widely used to evaluate the reliability and quality of YouTube videos referred to other disease conditions [31, 34, 37, 38], and is the most used method according to Betancourt et al. [44]. Discern consists of 15 items with two parts: reliability (items 1 to 8), and quality of information about treatment options (items 9 to 15). Each item is scored from 1 (low reliability/quality) to 5 points (high reliability/quality), and the average total score ranges from 15 (very poor) to 75 (excellent) [43].

### Statistical analysis

The SPSS version 27.0 statistical package was used to analyse the data. A descriptive statistical analysis was performed to quantify the characteristics of the videos in terms of frequencies and percentages. The Kolmogorov–Smirnov test was used to assess sample distribution; the results indicated non-normal distribution of the data. The Mann–Whitney *U*-test was used to identify differences between the video creation sources, and the Kruskal–Wallis test was used to compare video content between groups. Spearman’s correlation coefficient (rho) was used to analyse correlations between raters and between the characteristics of the videos and the Discern scores.

## Results

### Video characteristics

Of all the videos identified (*n* = 300), 35 were included in the analysis, with 28.6% (*n* = 10) being categorised as videos focused on combined prevention and treatment, and 82.9% (*n* = 29) were made by healthcare or academic professionals. The video characteristics are described in Table 1 and in supplementary material 1.

**Table 1:** Video characteristics

	Median (RI)
Likes	71 (555)
Comments	3 (15)
Views	7276 (25,875)
Length (minutes)	9.52 (12.52)
Likes ratio	100 (0)
View ratio	4.38 (28.22)
VPI	3.43 (28.85)
Days online	1072 (1514)
Video content	% (n)
Informative	25.7 (9)
Preventive	25.7 (9)
Treatment	14.3 (5)
Informative and preventive	5.7 (2)
Preventive and treatment	28.6 (10)
Video source	
Healthcare or academic professionals	82.9 (29)
Others	17.1 (6)

### Video quality and reliability assessment

The median scores of the two evaluators showed significant and strong correlations in terms of reliability (*r* = 0.896; *p* < 0.001), quality (*r* = 0.911; *p* < 0.001) and the total Discern score (*r* = 0.872; *p* < 0.001).

Videos made by healthcare or academic professionals had a significantly higher reliability score (*p* < 0.017) and Discern total score (*p* < 0.03) than those made by others. No significant differences were observed in other video characteristics based on the source of production between videos made by healthcare or academic professionals and by others (Table 2).

**Table 2:** Comparison of video characteristics according to source of production

	Median (RI)			
	Total (*n* = 35)	Healthcare or academic professionals (*n* = 29)	Other sources (*n* = 6)	*P*
Discern				
Reliability (1–8)	2. 6 (0.44)	2.6 (0.35)	2.1 (0.50)	0.017
Quality (9–15)	1.4 (0.5)	1.5 (0.39)	1.2 (0.52)	0.145
Discern score	30.5 (7.00)	31.0 (6.25)	25.5 (4.88)	0.03
Likes	71 (555)	86 (646.51)	34 (90.25)	0.076
Video length	9.5 (12.52)	9.5 (12.98)	9.4 (20.46)	0.569
Views	7276 (25,875)	10,762 (51,224)	1087.5 (7883.75)	0.066
Comments	3 (15)	3 (20.50)	4 (8.25)	0.623
VPI	3.4 (28.85)	5.7 (44.29)	1.8 (3.30)	0.088

There were no significant differences in reliability and quality and total Discern score based on the content of the videos between videos made by healthcare or academic professionals and by others (Table 3).

**Table 3:** Comparison of quality, reliability and total Discern score of the videos based on content

	Median (RI)					
	Informative (*n* = 9)	Preventive (*n* = 9)	Treatment (*n* = 5)	Informative and preventive (*n* = 2)	Preventive and treatment (*n* = 10)	*p*
Discern	30.5 (13.50)	29 (3.75)	30.5 (5)	31.75 (-)	31.3 (9.25)	0.856
Reliability	2.4 (1.06)	2.5 (0.09)	2.5 (0.44)	2.7 (-)	2.7 (0.58)	0.795
Quality	1.6 (1.04)	1.3 (0.39)	1.4 (0.25)	1.5 (-)	1.6 (0.75)	0.496

### Correlation between VPI and Discern scores

An important and significant correlation was observed between the comments assessed with the VPI (*r* = 0.472; *p* = 0.004) and the number of likes (*r* = 0.407; *p* = 0.015). Likewise, a significant and large effect was observed between the duration of the videos and reliability (*r* = 0.472; *p* = 0.004), quality (*r* = 0.469; *p* = 0.004) and the total Discern score (*r* = 0.476; *p* = 0.004) (Table 4).

**Table 4:** Correlation between the video characteristics and Discern scores

		Discern	Reliability	Quality	Comments	Length
VPI	*r* *p*	0.124 0.477	0.213 0.219	0.002 0.989	0.472 0.004	− 0.150 0.390
Likes	*r* *p*	0.054 0.758	0.143 0.413	− 0.056 0.750	0.407 0.015	− 0.196 0.259
Comments	*r* *p*	0.084 0.631	0.172 0.322	0.094 0.591	-	
Views	*r* *p*	0.079 0.653	0.167 0.3383	− 0.035 0.843		
Length	*r* *p*	0.485 0.003	0.400 0.017	0.497 0.002		-

## Discussion

The findings of this study demonstrated that videos made by healthcare and academic professionals had higher reliability, quality and overall Discern score than those made by individuals (e.g., personal experience). YouTube is a platform widely used by the general population to access health-related information [19]. However, the results of this study suggest that there is clear heterogeneity in terms of quality and reliability depending on the source of content. Such heterogeneity could be due to the lack of a standardised method for content evaluation prior to video release [24]. The lack of standardised evaluation for videos related to disease prevention and treatment (e.g., lymphedema) on YouTube raises concern about the potential harmful impact of low-quality videos and misleading contents on patients seeking information from YouTube [22]. Whilst accurate and adequate information can contribute to improve the capacity of individuals to change or form healthy behaviours [9], given that zero risk does not exist, the access to inadequate information or misinformation could result in undesirable or even harmful effects. For example, the adoption of inappropriate health-related decisions based on erroneous or inaccurate information [27] causes delays in the provision of care or in seeking medical attention, as well as misallocation of health resources [45]. Therefore, as suggested by O'Rourke et al. [46] and Madathil et al. [22], it is essential to carry out an adequate assessment of digital resources, focusing on the content of YouTube videos that provide information regarding disease information and related health behaviours.

Lymphedema prevention, independently or in conjunction with treatment options, was addressed by approximately 50% of the videos analysed. This result differs from the data found in previous studies, where informative content about lymphedema diagnosis, symptoms or treatment predominated over other contents [32, 33, 37]. Nevertheless, the results of our study, from a salutogenic point of view, are essential for healthcare providers and patients, as lymphedema is currently incurable and produces a long-term and debilitating effect upon the functional [47] and psychological [48] well-being of breast cancer survivors, as well as on the quality of their social relationships [49]. Thus, videos that provide accurate information regarding lymphedema prevention may help patients, but misleading information may bring more harm to patients.

Our study found that most of the videos on breast cancer-related lymphedema were made by healthcare or academic professionals, which is common for YouTube videos related to other health issues [32, 34, 37, 42]. Similarly to videos on other health issues [23, 33, 36, 43], our study also found that videos on breast cancer-related lymphedema made by healthcare or academic professionals yielded significantly higher scores in terms of quality and reliability. It is worth highlighting the study carried out by Küçükakkaş et al. [37], who evaluated English language videos on lymphedema rehabilitation after breast cancer. This study also found higher reliability scores for educational videos made by health professionals. High quality and reliability videos made by healthcare or academic professionals reflect expert knowledge and clinical experience needed for creating educational videos regarding health issues, such as lymphedema prevention and treatment. Clinical practice should include patient education as part of routine care; in this regard, emphasis should be placed on the importance of the source of production of the videos, prioritising the selection of those made by healthcare or academic professionals.

An important finding in this study was the significant correlation between VPI and the number of comments and non-correlation with the Discern scores—these being results previously observed by other researchers [38]. This finding raises concerns regarding the value of VPI as an indicator of quality. Perhaps it is important to limit its use and the elements that constitute it, such as likes and views on videos regarding disease and health.

Another finding consistent with the existing literature was the positive correlation between the quality and reliability of the videos and their duration [33, 37, 39]. Shorter videos may include less detailed information than longer videos. However, it is important to highlight that we live in a culture of immediacy, which translates into the need to obtain a quick response amongst people between 25 and 54 years of age, who are the main users of YouTube [20]. This culture of immediacy presents important and difficult challenges for future video productions. Therefore, future videos on lymphedema should be critically evaluated before being hosted on the platform, and/or the people who make the videos should consult experts and pay special attention to the quality and reliability of the videos [43, 46].

## Limitations

Firstly, the YouTube platform changes over time, and new videos are uploaded every day without evaluation. However, our study provided fundamental criteria for patients and healthcare professionals to identify quality videos on lymphedema based on their personalised needs. The dynamic nature of YouTube reinforces the importance of the continuous evaluation of YouTube videos related to health issues (e.g., lymphedema), and other digital resources. Secondly, YouTube policy sorts videos according to its rating system and displays personalised results based on the search history and views; thus, the results could differ depending on how people perform the searches. However, the consistency of the results of our study with those found in previous publications highlights the need to ensure the rigour, quality and reliability of the health-related videos hosted on this platform.

## Conclusions

Higher reliability and quality scores were found in Spanish language YouTube videos on breast cancer-related lymphedema made by healthcare or academic professionals. This finding provides a strong rationale for educating breast cancer survivors searching for lymphedema information to select videos made by healthcare or academic professionals. Given the heterogeneity of content, quality and reliability regarding the breast cancer-related lymphedema videos hosted on YouTube, it is essential to perform a standardised evaluation prior to video publication in order to ensure that the breast cancer survivors, as end-users, receive accurate and quality information from YouTube.

### Supplementary Information

Below is the link to the electronic supplementary material.

Supplementary file1 (DOCX 17 KB)

## Data Availability

No datasets were generated or analysed during the current study.

## References

[1] World Health Organization. Breast cancer [Internet]. Geneve: Available from: https://www.who.int/news-room/fact-sheets/detail/breast-cancer. Accessed April 16, 2024.

[2] DiSipio T, Rye S, Newman B, Hayes S (2013) Incidence of unilateral arm lymphoedema after breast cancer: a systematic review and meta-analysis. Lancet Oncol 14(6):500–515. 10.1016/S1470-2045(13)70076-7 23540561 10.1016/S1470-2045(13)70076-7

[3] Jinbo K, Fujita T, Kasahara R, Jinbo R, Kisara S, Onobe J et al (2023) The effect of combined risk factors on breast cancer-related lymphedema: a study using decision trees. Breast Cancer 30(4):685–688. 10.1007/s12282-023-01450-9 36917351 10.1007/s12282-023-01450-9

[4] Rupp J, Hadamitzky C, Henkenberens C, Christiansen H, Steinmann D, Bruns F (2019) Frequency and risk factors for arm lymphedema after multimodal breast conserving treatment of nodal positive breast Cancer – a long-term observation. Radiat Oncol 14(1):39. 10.1186/s13014-019-1243-y 30845971 10.1186/s13014-019-1243-y PMC6407279

[5] Togawa K, Ma H, Smith AW, Neuhouser ML, George SM, Baumgartner KB et al (2021) Self-reported symptoms of arm lymphedema and health-related quality of life among female breast cancer survivors. Sci Rep 11(1):10701. 10.1038/s41598-021-89055-0 34021179 10.1038/s41598-021-89055-0 PMC8139966

[6] Martin-Payo R, Cachero-Rodriguez J, Alvarez-Gomez E, Fu MR, Llaneza-Folgueras A, Fernandez-Alvarez M (2022) Data for the Spanish adaptation of Breast Cancer and Lymphedema Symptom Experience Index (BCLE SEI Esp). Data Brief 45:108699. 10.1016/j.dib.2022.108699 36426092 10.1016/j.dib.2022.108699 PMC9679694

[7] Fu MR, Axelrod D, Cleland CM, Qiu Z, Guth AA, Kleinman R et al (2015) Symptom report in detecting breast cancer-related lymphedema. Breast Cancer 7:345–352. 10.2147/BCTT.S87854 26527899 10.2147/BCTT.S87854 PMC4621182

[8] Deveci Z, Karayurt Ö, Eyugör S (2021) Self – care practices, patient education in women with breast cancer – related lymphedema. Turk J Phys Med Rehabil 67(2):187–195. 10.5606/tftrd.2021.5022 34396069 10.5606/tftrd.2021.5022 PMC8343163

[9] Perdomo M, Davies C, Levenhagen K, Ryans K, Gilchrist L (2023) Patient education for breast cancer – related lymphedema: a systematic review. J Cancer Surviv. 17:1–15. 10.1007/s11764-022-01262-4 36207626 10.1007/s11764-022-01262-4 PMC9546750

[10] Changizi M, Ghahremani L, Ahmadloo N, Kaveh MH (2022) The patient health engagement model in cancer management: effect of physical activity, distress management, and social support intervention to improve the quality of life in breast cancer patients. Int J Breast Cancer 2022:1944852. 10.1155/2022/1944852 35535128 10.1155/2022/1944852 PMC9078844

[11] Cansız G, Arıkan Dönmez A, Kapucu S, Borman P (2022) The effect of a self-management lymphedema education program on lymphedema, lymphedema-related symptoms, patient compliance, daily living activities and patient activation in patients with breast cancer-related lymphedema: a quasi-experimental study. Eur J Oncol Nurs 56:102081. 10.1016/j.ejon.2021.102081 34875398 10.1016/j.ejon.2021.102081

[12] Omidi Z, Kheirkhah M, Abolghasemi J, Haghighat S (2020) Effect of lymphedema self –management group – based education compared with social network – based education on quality of life and fear of cancer recurrence in women with breast cancer: a randomized controlled clinical trial. Qual Life Res 29(7):139–146. 10.1007/s11136-020-02455-z 10.1007/s11136-020-02455-z PMC7295820 32152817

[13] Michie S, van Stralem MM, West R (2011) The behaviour change wheel: a new method for characterizing and designing behaviour change interventions. Implement Sci 6:42. 10.1186/1748-5908-6-42 21513547 10.1186/1748-5908-6-42 PMC3096582

[14] Borman P, Yaman A, Yasrebi S, Özdemir O (2017) The importance of awareness and education in patients with breast cancer – related lymphedema. J Cancer Educ 32:629–633. 10.1007/s13187-016-1026-1 27048148 10.1007/s13187-016-1026-1

[15] Pervane Vural S, Ayhan FF, Soran A (2020) The role of patient awareness and knowledge in developing secondary lymphedema after breast and gynecologic cancer surgery. Lymphat Res Biol 18(6):526–533. 10.1089/lrb.2020.0059 33026963 10.1089/lrb.2020.0059

[16] Buki LP, Rivera-Ramos ZA, Kanagui-Muñoz M, Heppner PP, Ojeda L, Lehardy EN et al (2021) “I never heard anything about it”: knowledge and psychosocial needs of Latina breast cancer survivors with lymphedema. Womens Health (Lond) 17:17455065211002488. 10.1177/17455065211002488 33764235 10.1177/17455065211002488 PMC8010798

[17] Marin-Torres V, Valverde Aliaga J, Sánchez Miró I, Del Castillo S, Vicente MI, Polentinos-Castro E, Garrido BA (2013) Internet as an information source for health in primary care patients and its influence on the physician-patient relationship. Aten Primaria 45(1):46–53. 10.1016/j.aprim.2012.09.004 23140836 10.1016/j.aprim.2012.09.004 PMC6985490

[18] Latif MZ, Hussain I, Saeed R, Qureshi MA, Maqsood U (2019) Use of smart phones and social media in medical education: trends, advantages, challenges and barriers. Acta Inform Med 27(2):133–138. 10.5455/aim.2019.27.133-138 31452573 10.5455/aim.2019.27.133-138 PMC6688444

[19] Han CJ, Lee YJ, Demiris G (2018) Interventions using social media for cancer prevention and management: a systematic review. Cancer Nurs 41(6):E19–E31. 10.1097/NCC.0000000000000534 28753192 10.1097/NCC.0000000000000534 PMC5787052

[20] Global Media Insight. YouTube user statistics 2023 [Internet]. GMI Blogger. Available from: https://www.globalmediainsight.com/blog/youtubeusers-statistics Accessed July 15, 2023.

[21] Rapp AK, Healy MG, Charlton ME, Keith JN, Rosenbaum ME, Kapadia MR (2016) YouTube is the most frequently used educational video source for surgical preparation. J Surg Educ 73(6):1072–1076. 10.1016/j.jsurg.2016.04.024 27316383 10.1016/j.jsurg.2016.04.024 PMC7263439

[22] Madathil KC, Rivera-Rodríguez AJ, Greenstein JS, Gramopadhye AK (2015) Healthcare information on YouTube: a systematic review. Health Informatics J 21(3):173–194. 10.1177/1460458213512220 24670899 10.1177/1460458213512220

[23] Helming AG, Adler DS, Keltner C, Igelman AD, Woodworth GE (2021) The content quality of youtube videos for professional medical education: a systematic review. Acad Med 96(10):1484–1493. 10.1097/ACM.0000000000004121 33856363 10.1097/ACM.0000000000004121

[24] Drozd B, Couvillon E, Suarez A (2018) Medical YouTube videos and methods of evaluation: literature review. JMIR Med Educ 4(1):e3. 10.2196/mededu.8527 29434018 10.2196/mededu.8527 PMC5826977

[25] Chan KS, Shelat VG (2021) We asked the experts: emerging role of YouTube surgical videos in education and training. World J Surg 45(2):417–419. 10.1007/s00268-020-05660-6 32591845 10.1007/s00268-020-05660-6

[26] Osman W, Mohamed F, Elhassan M, Shoufan A (2022) Is YouTube a reliable source of health-related information? A systematic review. BMC Med Educ 22(1):382. 10.1186/s12909-022-03446-z 35590410 10.1186/s12909-022-03446-z PMC9117585

[27] Luo Y, Maafs-Rodríguez AG, Hatfield DP (2024) The individual-level effects of social media campaigns related to healthy eating, physical activity, and healthy weight: a narrative review. Obes Sci Pract 10(1):e731. 10.1002/osp4.731 38187123 10.1002/osp4.731 PMC10767147

[28] Wu R, Huang X, Dong X, Zhang H, Zhuang L (2019) Obese patients have higher risk of breast cancer-related lymphedema than overweight patients after breast cancer: a meta-analysis. Ann Transl Med. 7(8):172. 10.21037/atm.2019.03.44 31168453 10.21037/atm.2019.03.44 PMC6526274

[29] Martínez-Jaimez P, Fuster Linares P, Piller N, Masia J, Yamamoto T, López-Montoya L et al (2022) Multidisciplinary preventive intervention for breast cancer-related lymphedema: an international consensus. Eur J Cancer Care 31(6):e13704. 10.1111/ecc.13704 10.1111/ecc.13704 36113999

[30] Singh AG, Singh S, Singh PP (2012) YouTube for information on rheumatoid arthritis-a wakeup call? J Rheumatol 39(5):899–903. 10.3899/jrheum.111114 22467934 10.3899/jrheum.111114

[31] Ayoub G, Chalhoub E, Sleilaty G, Kourie HR (2021) YouTube as a source of information on breast cancer in the Arab world. Support Care Cancer 29(12):8009–8017. 10.1007/s00520-021-06403-6 34224018 10.1007/s00520-021-06403-6

[32] Ozsoy-Unubol T, Alanbay-Yagci E (2021) YouTube as a source of information on fibromyalgia. Int J Rheum Dis 24(2):197–202. 10.1111/1756-185X.14043 33355406 10.1111/1756-185X.14043

[33] Lee KN, Tak HJ, Park SY, Park ST, Park SH (2022) YouTube as a source of information and education on endometriosis. Medicine (Baltimore) 101(38):e30639. 10.1097/MD.0000000000030639 36197187 10.1097/MD.0000000000030639 PMC9509122

[34] Güloğlu S, Özdemir Y, Basim P, Tolu S (2022) YouTube English videos as a source of information on arm and shoulder exercise after breast cancer surgery. Eur J Cancer Care (Engl) 31(6):e13685. 10.1111/ecc.13685 35970600 10.1111/ecc.13685

[35] Yurdaisik I (2020) Analysis of the most viewed first 50 videos on YouTube about breast cancer. Biomed Res Int 2020:2750148. 10.1155/2020/2750148 32596288 10.1155/2020/2750148 PMC7273466

[36] Bai G, Pan X, Zhao T, Chen X, Liu G, Fu W (2022) Quality assessment of YouTube videos as an information source for testicular torsion. Front Public Health 10:905609. 10.3389/fpubh.2022.905609 35664123 10.3389/fpubh.2022.905609 PMC9157819

[37] Küçükakkaş O, İnce B (2022) Can YouTube be used as an educational tool in lymphedema rehabilitation? Arch Physiother 12(1):5. 10.1186/s40945-022-00130-9 35236412 10.1186/s40945-022-00130-9 PMC8890817

[38] Zhang X, Yang Y, Shen YW, Zhang KR, Ma LT, Ding C et al (2022) Quality of online video resources concerning patient education for neck pain: a YouTube-based quality-control study. Front Public Health 10:972348. 10.3389/fpubh.2022.972348 36211682 10.3389/fpubh.2022.972348 PMC9533122

[39] Norgaard O, Furstrand D, Klokker L, Karnoe A, Osborne RH (2015) The e-health literacy framework: a conceptual framework for characterizing e-health users and their interaction with e-health systems. Knowl Manag E-Learn 7:522–40. 10.34105/j.kmel.2015.07.035 10.34105/j.kmel.2015.07.035

[40] World Population Review. Spanish speaking countries in 2024 [Internet]. California: Available from: https://worldpopulationreview.com/country-rankings/spanish-speaking-countries. Accessed on April 26, 2024.

[41] YouTube. Búsqueda de YouTube [Internet]. California: Available from: https://www.youtube.com/howyoutubeworks/productfeatures/search/ Accessed July 15, 2023.

[42] Erdem MN, Karaca S (2018) Evaluating the accuracy and quality of the information in kyphosis videos shared on YouTube. Spine 43(22):E1334–E1339. 10.1097/BRS.0000000000002691 29664816 10.1097/BRS.0000000000002691

[43] Charnock D, Shepperd S, Needham Gann R (1999) DISCERN: an instrument for judging the quality of written consumer health information on treatment choices. J Epidemiol Commun Health. 53(2):105–11. 10.1136/jech.53.2.105 10.1136/jech.53.2.105 PMC1756830 10396471

[44] Betancourt A, Campillo N, Mieres C (2021) Información sobre la salud: una revisión de la literatura existente sobre YouTube como fuente de información sanitaria. Rev Esp Com Sal. 11(1):1–18. 10.35669/rcys.2021.11.e207 10.35669/rcys.2021.11.e207

[45] do IJ, Borges, Nascimento, Pizarro AB, Almeida JM, Azzopardi-Muscat N, Gonçalves MA, Björklund M et al (2022) Infodemics and health misinformation: a systematic review of reviews. Bull World Health Organ 100(9):544–61. 10.2471/BLT.21.287654 36062247 10.2471/BLT.21.287654 PMC9421549

[46] O’Rourke B, Oortwijn W, Schuller T, International Joint Task Group (2020) The new definition of health technology assessment: a milestone in international collaboration. Int J Technol Assess Health Care. 36(3):187–90. 10.1017/S0266462320000215 32398176 10.1017/S0266462320000215

[47] Park JH, Merriman J, Brody A, Fletcher J, Yu G, Ko E et al (2021) Limb volume changes and activities of daily living: a prospective study. Lymphat Res Biol 19(3):261–268. 10.1089/lrb.2020.0077 33185515 10.1089/lrb.2020.0077 PMC8220540

[48] Fu MR, Ridner SH, Hu SH, Stewart BR, Cormier JN, Armer JM (2013) Psychosocial impact of lymphedema: a systematic review of literature from 2004 to 2011. Psychooncology 22(7):1466–1484. 10.1002/pon.3201 23044512 10.1002/pon.3201 PMC4153404

[49] Fu X, Lu Q, Pang D, Shen A, Shih YA, Wei X (2023) Experiences of breast cancer survivors with lymphedema self-management: a systematic review of qualitative studies. J Cancer Surviv 17(3):619–633. 10.1007/s11764-022-01225-9 35773611 10.1007/s11764-022-01225-9

