# Supplementary material for: YouTube videos on lymphedema as an information source for Spanish speaking breast cancer survivors
Source: Support Care Cancer. 2024 Jul 24;32(8):540. doi: 10.1007/s00520-024-08746-2 (PMC11266410; doi:10.1007/s00520-024-08746-2)
Supplement: Supplementary file 1 — Supplementary file1 (DOCX 17 KB) [file 520_2024_8746_MOESM1_ESM.docx]

Supplementary material 1. Video content information and link

|  | **Link** | **Content** |
| --- | --- | --- |
| 1 | <https://www.youtube.com/watch?v=9PUg_6AzDHs&t=13s> | Preventive |
| 2 | <https://www.youtube.com/watch?v=2SgQEQK7eAM&t=2s> | Informative |
| 3 | <https://www.youtube.com/watch?v=7TnZ3RtrTMo> | Preventive |
| 4 | <https://www.youtube.com/watch?v=6-Myoh0NdnA> | Preventive/Treatment |
| 5 | <https://www.youtube.com/watch?v=bjMKhySMPzw&t=6s> | Treatment |
| 6 | <https://www.youtube.com/watch?v=7RrYk27E2Oo> | Preventive |
| 7 | <https://youtu.be/EXht7740A7g> | Treatment |
| 8 | <https://www.youtube.com/watch?v=NSZLrN-1wQ4&t=2s> | Preventive/Treatment |
| 9 | <https://youtu.be/eTTpSo1mAE0> | Informative |
| 10 | <https://youtu.be/gcERAuOlJVs> | Informative |
| 11 | <https://www.youtube.com/watch?v=Lr5T2Lphdos> | Treatment |
| 12 | <https://www.youtube.com/watch?v=eOCA8X6mVdg&t=3s> | Preventive/Treatment |
| 13 | <https://youtu.be/Nmbc-du7IlY> | Preventive |
| 14 | <https://www.youtube.com/watch?v=jzl5HrWVtNM&t=3s> | Preventive |
| 15 | <https://www.youtube.com/watch?v=Xh6FOckPFHY> | Preventive/Treatment |
| 16 | <https://www.youtube.com/watch?v=whFuP0psvA4&t=6s> | Preventive |
| 17 | <https://www.youtube.com/watch?v=Scl225v6WPM> | Preventive/Treatment |
| 18 | <https://www.youtube.com/watch?v=ThtosSbY9zk> | Preventive/Treatment |
| 19 | <https://youtu.be/XQr5d-5YhRk> | Informative |
| 20 | <https://www.youtube.com/watch?v=7C6hNBrbLXQ> | Preventive /Treatment |
| 21 | <https://www.youtube.com/watch?v=RUYZEbKGGHQ&t=7s> | Preventive/Treatment |
| 22 | <https://www.youtube.com/watch?v=bYEKejL2gk0&t=2s> | Preventive/Treatment |
| 23 | <https://www.youtube.com/watch?v=S5mz3dZ04MU> | Informative |
| 24 | <https://www.youtube.com/watch?v=FIrdT8Css8U> | Informative |
| 25 | <https://www.youtube.com/watch?v=cnopkLlN3i8&t=3s> | Informative |
| 26 | <https://youtu.be/JVJe0E0Gf5s> | Informative |
| 27 | <https://www.youtube.com/watch?v=rvV31VZI5rI&t=3s> | Preventive |
| 28 | <https://youtu.be/5K8kO5d6fnY> | Preventive/Treatment |
| 29 | <https://www.youtube.com/watch?v=4Je7PFxIBh0> | Preventive |
| 30 | <https://www.youtube.com/watch?v=wraboR9_iSg&t=5s> | Preventive |
| 31 | <https://www.youtube.com/watch?v=_0wp58H5OxQ&t=3s> | Informative/Preventive |
| 32 | <https://youtu.be/IaerQawo1Gw> | Treatment |
| 33 | <https://www.youtube.com/watch?v=GyDvJB_0LGQ&t=3s> | Treatment |
| 34 | <https://youtu.be/YRV-dQp3FYc> | Informative |
| 35 | <https://www.youtube.com/watch?v=srLQqHyku_M&t=3s> | Informative/Preventive |
